# Determinants of Malaria Program Expenditures during Elimination: Case Study Evidence from Select Provinces in the Philippines

**DOI:** 10.1371/journal.pone.0073352

**Published:** 2013-09-27

**Authors:** Jenny X. Liu, Gretchen Newby, Aprielle Brackery, Cara Smith Gueye, Christine J. Candari, Luz R. Escubil, Lasse S. Vestergaard, Mario Baquilod

**Affiliations:** 1 The Global Health Group, University of California San Francisco, San Francisco, California, United States of America; 2 Mailman School of Public Health, Columbia University, New York, New York, United States of America; 3 Department of Health, Government of the Philippines, Manila, Philippines; 4 Independent Consultant, Manila, Philippines; 5 Philippines Country Office, World Health Organization, Manila, Philippines; Mahidol-Oxford Tropical Medicine Research Unit, Thailand

## Abstract

...Even though eliminating malaria from the endemic margins is a part of the Global Malaria Action Plan, little guidance exists on what resources are needed to transition from controlling malaria to eliminating it. Using Philippines as an example, this study aimed to (1) estimate the financial resources used by sub-national malaria programs in different phases during elimination and (2) understand how different environmental and organizational factors may influence expenditure levels and spending proportions. The Philippines provides an opportunity to study variations in sub-national programs because its epidemiological and ecological diversity, devolved health system, and progressive elimination strategy all allow greater flexibility for lower-level governments to direct activities, but also create challenges for coordination and resource mobilization. Through key informant interviews and archival record retrieval in four selected provinces chosen based on eco-epidemiological variation, expenditures associated with provincial malaria programs were collected for selected years (mid-1990s to 2010). Results show that expenditures per person at risk per year decrease as programs progress from a state of controlled low-endemic malaria to elimination to prevention of reintroduction regardless of whether elimination was deliberately planned. However, wide variation across provinces were found: expenditures were generally higher if mainly financed with donor grants, but were moderated by the level of economic development, the level of malaria transmission and receptivity, and the capacity of program staff. Across all provinces, strong leadership appears to be a necessary condition for maintaining progress and is vital in controlling outbreaks. While sampled provinces and years may not be representative of other sub-national malaria programs, these findings suggest that the marginal yearly cost declines with each phase during elimination.

## Introduction

In recent years, considerable progress has been made toward reducing the global morbidity and mortality from malaria [1]. Massive scale-up of malaria interventions—insecticide-treated nets (ITNs), indoor residual spraying (IRS), and increased access to diagnostics and effective treatment—in addition to economic development and improved general health care, have helped to reduce transmission in many countries [1,2]. Many countries now have a goal of national or subnational malaria elimination, defined as the interruption of transmission in a specified geographic area resulting in zero locally contracted cases [3].

Even though eliminating malaria from the endemic margins is a part of the Global Malaria Action Plan [4], little guidance exists on what resources are needed to transition from controlling malaria to eliminating it. Little is known about intervention mix needed to eliminate transmission once a state of controlled-low endemic malaria (CLM) has been achieved, or when malaria is no longer a major public health threat [5]. Even less is known about the financial inputs needed to reposition a program from control to elimination and prevention of reintroduction (POR), despite the fact that many countries will be confronted with these programmatic decisions as malaria further declines.

To understand the financial resources needed for malaria elimination, a retrospective case study was conducted of provincial malaria programs in the Philippines. In selected provinces with diverse eco-epidemiological environments, the aim was to document the interventions used at various stages of elimination and their costs, and determine the marginal cost per year between phases of elimination. Variations in expenditures per year across provinces were compared in relation to malaria burden levels and local epidemiology, geography and economic development in the area, sources of program financing, organizational structure and capacity, and the degree to which malaria elimination was an active pursuit. Table 1 summarizes the key findings of the study. Along with broader implications for national malaria control programs in other countries, insights for furthering the Philippines’ sub-national elimination strategy are discussed.

**Table 1:** Summary of Key Findings.

**Expenditure determinants**	**Cross-province trends**
Malaria burden, eco-epidemiology	• Less active intervention needed in places where burden is historically low
Geography, economic development	• Higher expenditures needed to reach vulnerable groups located in remote places
	• Expenditures moderated by pace of development
Sources of financing	• External funding encourages more spending on M&E, extra commodities
	• More “prudent” spending seen with tighter budget constraints, but potentially limited capacity for emergency response
Organizational structure and capacity	• Strong leadership and local government buy-in and support essential for successful program implementation
	• Devolution/decentralization of malaria removed experienced national staffers from programs, lowered morale, left provincial programs vulnerable in event of outbreak
Progress toward elimination	• Costs decrease as programs progress from state of controlled low endemicity to elimination to prevention of reintroduction
	• Decrease in costs seen regardless of whether elimination was actively pursued or passive result of external factors

### Background on malaria control in the Philippines

The Philippines has recently experienced a substantial reduction in annual malaria caseload, from over 36,000 indigenous cases in 2000 to fewer than 15,000 in 2011. As of 2012, 25% of the country’s population was living in malaria-free areas (areas with zero indigenous cases, or cases that do not to have a local origin), with another 73% residing in areas of low transmission (<1 case per 1,000 population), and the remaining 2% resides in areas of high transmission (>1 case per 1,000 population) [6]. The National Malaria Control Program (NMCP) aims to achieve zero malaria deaths to zero by 2014 and countrywide malaria elimination by 2020 [7].

**Figure 1:**
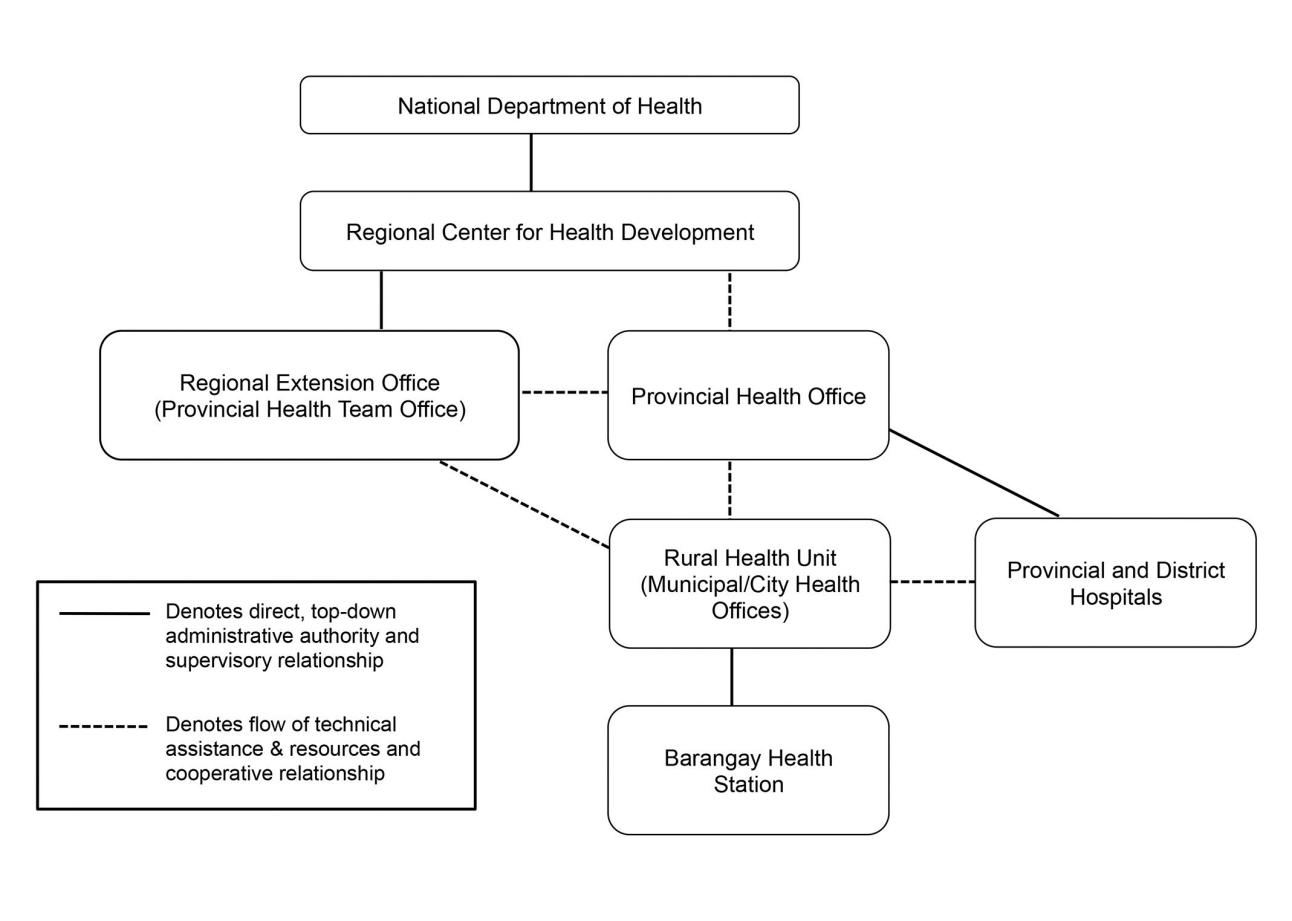
Philippines Health System Organization. Under the decentralized, or devolved, health system, technical assistance, policies, and guidelines for malaria control disseminate from the National Department of Health (DOH) to the Provincial Health Offices via Regional Centers for Health Development (CHD) and DOH representatives positioned in extension offices at the provincial level. Information and technical assistance is further propagated by provincial health office staff who conduct trainings and oversee malaria control activities at the municipal and *barangay* levels with the participation of DOH representatives. While some funding does flow downward from national, every government unit is expected to provide financial support for malaria activities occurring at its respective level. The Provincial Health Office serves as point of entry for external funding organizations, including Global Fund.

With over 7,000 islands in the Philippines, each unique in geography, ecology, sociopolitical situation, and malaria epidemiology, a national strategy of progressive malaria elimination allows local programs to custom-tailor interventions to each province’s needs, supported by a certification process to verify the achievement of elimination in each. Further, the public health system is highly devolved in which local government units (LGUs; includes provincial, municipal, and *barangay* or neighborhood/village levels) are tasked with program implementation. In 1993, the vertical malaria program under the National Department of Health (DOH) was decentralized, making provincial and municipal governments responsible for managing their own programs and financing a portion of their activities, supplemented with DOH budget outlays (see Figure 1 for an organizational depiction). Thus far, LGU capacity to take on program responsibility varies widely, and program decisions can still come from the DOH where local health systems lack experience [8].

The Philippines has also experienced increases in population health and economic development in recent years. Gross domestic product per capita has doubled in the past decade, from US$966 in 2001 to US$2,370 billion in 2011. Concurrently, life expectancy at birth has increased from 66.9 years to 68.8 years, and infant mortality has declined from 28.4 deaths per 1000 live births to 20.2 deaths per 1000 live births [9].

**Figure 2:**
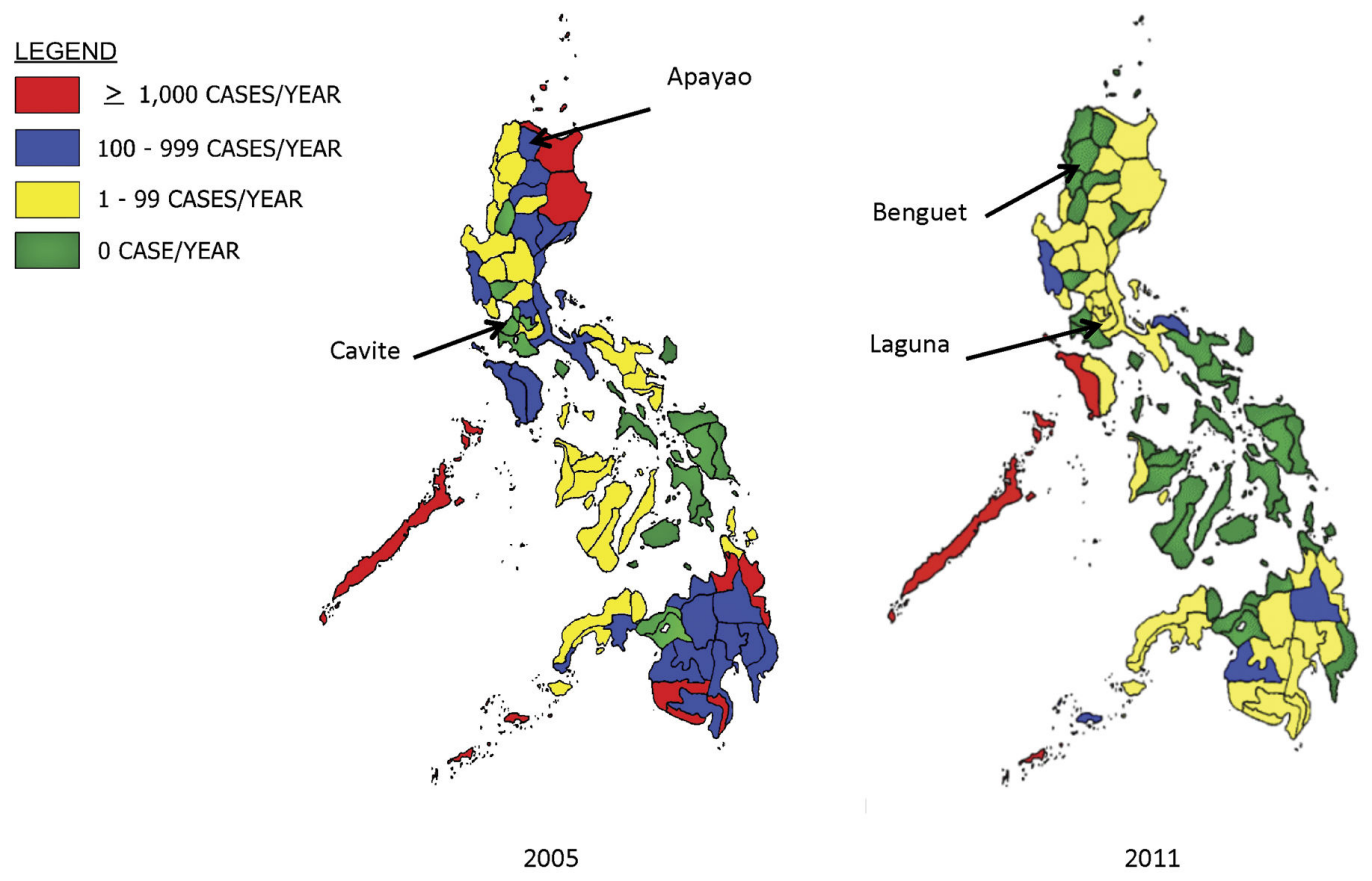
Malaria Burden Stratification in 2005 and 2011. Malaria burden across provinces in the Philippines are stratified based on the number of indigenous cases reported each year. Between 2005 and 2011, malaria cases have declined in many provinces. In the selected case study provinces, cases have declined in Apayao, Benguet, and Laguna. Cavite are Benguet are certified to be malaria-free according to the Philippines subnational malaria elimination certification standards.

At the same time, malaria has been progressively eliminated. Figure 2 displays the decline in number of indigenous cases reported (confirmed and clinically diagnosed) between 2005 and 2011, and Table 2 defines the level of endemicity classifications across provinces and the number of provinces in each as of 2010. However, the sustainability of malaria program funding is currently threatened. Financial support for malaria from the Global Fund to Fight AIDS, Tuberculosis and Malaria (GFATM) may not be renewed beyond 2014 under revised grant allocation criteria. Therefore, an in-depth understanding of the experiences and financial resources used by provinces that have recently embarked on or achieved elimination is needed to inform future strategic planning.

**Table 2:** Stratification Scheme of Malaria Endemic Areas in the Philippines, 2010.

**Stratum**	**Definition**	**No. of Provinces**	**No. of** **Cities**
1. Stable Risk	With at least 1 *barangay* that has a continuous presence of at least one indigenous malaria case in a month for 6 months or more at any time during the past three years	29	8
1.1 high	w/> 1000 aver. malaria cases from 2007-2009	(5)	(2)
1.2 moderate	w/ 100 to <1000 average malaria cases from 2007-2009	(18)	(3)
1.3 low	with < 100 average malaria cases from 2007-2009	(6)	(3)
2. Unstable Risk	With at least 1 *barangay* that has a continuous presence of at least one indigenous malaria case in a month for less than 6 months at any time during the past three years	10	1
3. Epidemic Risk or Sporadic risk	With at least 1 *barangay* that has a presence of at least one indigenous malaria case at any time in the past 5 years	18	2
4. Malaria Free	Absence of indigenous malaria case for 5 past years even in the presence of malaria vector	23	-
Total		80	11

Source: [7]

Note: Figures in parentheses represent the number of provinces/cities that fall under the stable risk stratum.

## Methods

This case study employed a variety of qualitative approaches, including historical record review, key informant interviews, and extraction of expenditure data from program accounts.

### Ethics statement

Approval for this study was obtained from institutional review boards of the University of California, San Francisco (12-08953), Western Pacific Regional Office of the World Health Organization (2012.08.PHL.01.MVP), and the Philippines DOH (DREC201202).

### Sample selection

Four provinces on Luzon, the largest island in the Philippines archipelago, were chosen to represent a range of malaria eco-epidemiological environments and programs in different phases of malaria elimination. The selected provinces are denoted in Figure 2; in 2011, Benguet and Cavite were officially considered malaria-free while Apayao and Laguna were still progressing toward elimination. Because the NMCP shifted the national strategy from control to elimination in 2008 [7], provinces earlier declared malaria-free achieved this status without deliberately pursing elimination (i.e. Benguet and Cavite), whereas current provincial programs with low transmission are actively pursuing elimination as a goal (i.e. Apayao and Laguna).

Sampled years in each province were chosen based on the completeness of available records, representation of different program phases, and key events (e.g. an outbreak). The phases of elimination were defined according to a mix of criteria from WHO [3], Cohen et al. [5], and the Philippines DOH [7] where applicable:

Controlled low-endemic malaria (CLM): interventions have reduced endemic malaria transmission to such low levels that it does not constitute a major public health burden [5]; typically between <5 cases/1000 per population at risk per year (PPY) and 1 case/1000 PPY [3].

Elimination: interventions have interrupted endemic transmission and limited onward transmission from imported infections below a threshold at which risk of reestablishment is minimized [5]; typically when indigenous cases are below 1/1000 PPY [3].

Prevention of reintroduction: zero indigenous cases are maintained [3]; provinces are certified malaria-free if no indigenous cases arise for five consecutive years [7].

### Data collection

From June to September 2012, researchers went to selected provinces to conduct key informant interviews and retrieve records on program expenditures, intervention coverage, and epidemiological indicators. In each province, current and former health workers from government, hospitals, and private sector organizations with relevant experience and knowledge of the malaria program were identified as potential key informants. Interviews and data collection began at the regional level, followed by visits to provincial, municipal, and *barangay* health offices. Hospitals were also included if staff were involved in case management and diagnosis, but treatment and case follow-up are largely conducted by dedicated malaria personnel out of LGU health offices. A total of 54 key informants were interviewed.

After obtaining written informed consent, interviewers followed a semi-structured questionnaire, asking about the history of the malaria program, epidemiological trends, organizational structure, operations, and activities. At the end of each interview, key informants were asked to identify other individuals who would be knowledgeable about the covered topics. Interviews were conducted in either English or Tagalog and tape-recorded for transcription and translation. A second semi-structured questionnaire was followed to identify malaria program expenditures and financial records for program activities. To the extent possible, expenditure records on personnel, commodities, services, and capital equipment were collected for all malaria activities in the sampled years. Key indicators for malaria epidemiology, intervention coverage, and population data were collated from multiple sources and databases (i.e. the Field Health Service Information System, the Philippines Integrated Disease Surveillance and Response System, the Philippines Malaria Information System, and annual health reports, and operational plans). There were many gaps in epidemiological data, including indigenous and imported cases on national and provincial level, and intervention coverage information. All available data sources were accessed and triangulated when possible, but there may be some underreporting. For additional details on interview processes, transcript coding, and expenditure calculations, please see Appendix S1.

To account for differences in service delivery needs across provinces, total yearly expenditures are adjusted by the population at risk (PAR), defined as the total population residing within a *barangay* or sub-*barangay* unit that are not certified malaria-free (i.e. have had some amount of indigenous malaria cases in the past five years) per standard procedures of the DOH at the time. To ensure consistency, the geographic area units, boundaries, and definition used to identify PAR by the NMCP in the first selected sampled year for each province are applied to all subsequent years, adjusted for population growth if actual counts were not available.

### Data analysis

Interview transcriptions were analyzed using Atlas.ti 6.2. A coding scheme was developed to identify common themes, including ecology and environment, risk groups, program strategies and interventions, financial and human resources, challenges and success factors.

Expenditure data were organized and analyzed across three dimensions:

1. funding source: local (barangay and municipal), provincial, or national government, non-governmental and donor agencies, and in-kind contributions;
2. malaria activity: diagnosis and treatment, prevention and vector control, surveillance, information and education campaigns (IEC), and program management and monitoring and evaluation (M/M&E); and
3. expenditure type: personnel, commodities, services, and capital equipment.

Private individual and household expenditures were not captured. All expenditures were adjusted to 2010 prices and converted to US dollars.

Personnel data were further tabulated to construct a *staffing ratio* measure, calculated by dividing the raw count of malaria personnel by the full-time employee (FTE) equivalent (i.e. staffing ratio = number of reported people working on malaria / number needed if all employees were working full time). The staffing ratio thus reflects the degree of dispersion in time allocation across individuals. Hence, a low staff ratio of 2 would reflect a higher proportion of time spent on malaria per person across fewer people, or four people each spending 50% of their time on malaria, equivalent to two FTEs (i.e. 2= 4 people/2 FTE). Conversely, a high staff ratio of 10 would reflect a lower proportion of time spent on malaria per person across a relatively higher number people, or 20 people each spending 10% of their time on malaria, again equivalent to two FTEs (i.e. 10=20 people/2 FTE).

## Results

Findings for each province are presented below, followed by a comparison of results across provinces.

### Apayao province

**Figure 3:**
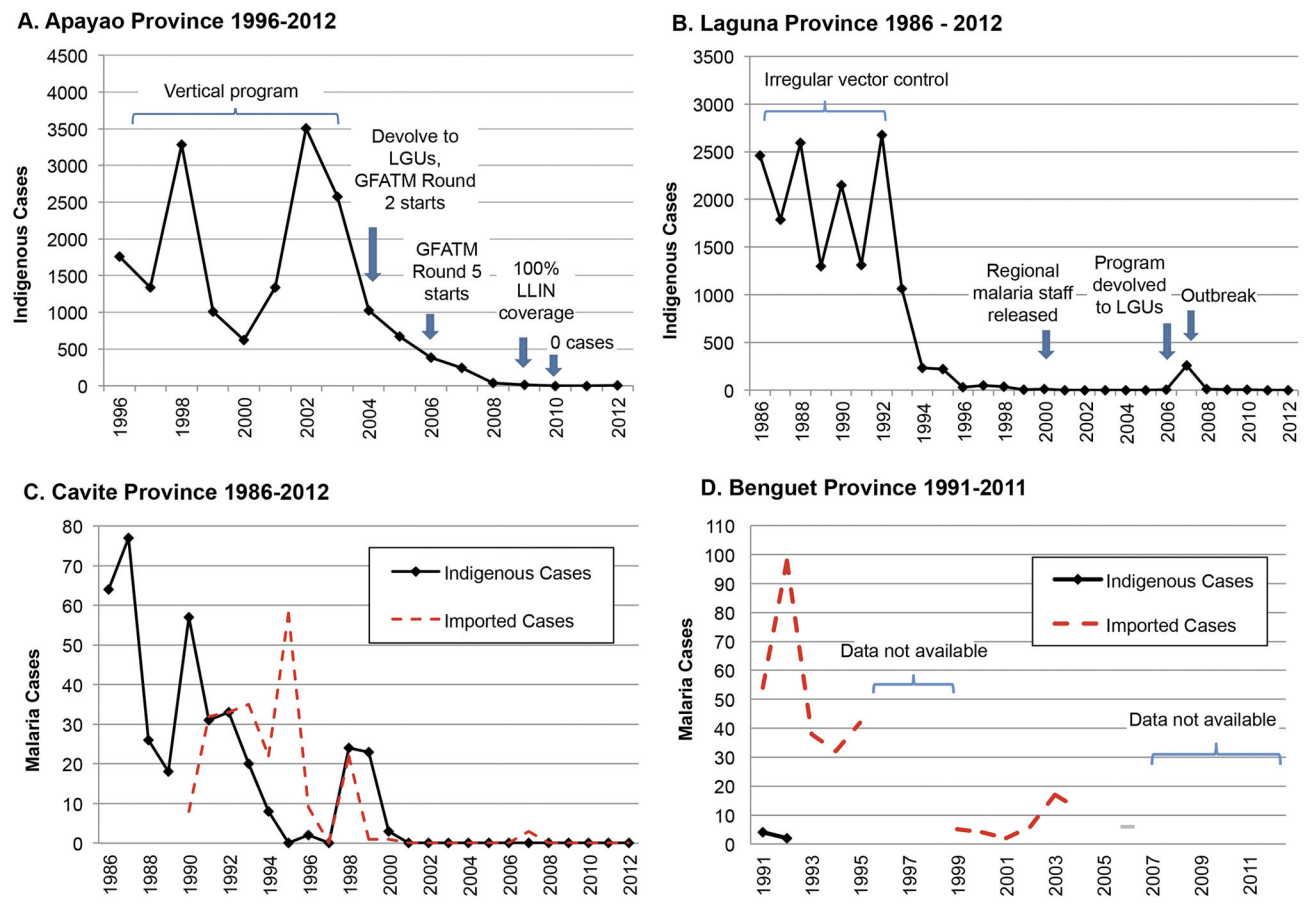
Malaria Cases for Selected Provinces of the Philippines. The number of indigenous and imported (when available) cases for each selected province is displayed, along with key programmatic events. Notes: (A) imported cases are not available; 100% long-lasting insecticide net (LLIN) coverage is defined as one net per 2.5 persons. (B) Imported cases are largely undocumented for this time period; the program was devolved to Local Government Units (LGUs) in 2006. (C) Cases from 1986-1999 were from both *P. vivax* and *P. falciparum*; all cases from 1999-2001 were from *P. vivax*. (D) Three cases occurred from July 2003-August 2004 that cannot be attributed to a single year and thus are not included; data are not available for 1996-1998, 2005, and 2007-2011. LGU = Local Government Unit.

All seven municipalities in Apayao are considered malaria endemic. Service delivery is more difficult given the mountainous terrain, limited infrastructure, and presence of highly mobile indigenous groups. Throughout the 1990s, annual caseload was in the thousands with peaks every four years (see Figure 3, Panel A). The malaria program was vertically structured until 2005, but insufficient resources from the DOH resulted in sporadic IRS. The program was devolved when funding from the GFATM in 2005 enabled capacity building for local staff and expanded access to care via microscopy centers established in remote *barangays*, staffed by trained, volunteer barangay health workers. Community volunteers assisted in IEC and stream-clearing activities, and IRS, ITN, and malaria drug distribution were all scaled up. The number of indigenous cases declined from a high of 3,503 (API (annual parasite index)=35.45 per 1000 PAR) in 2002 to 246 (API=2.37 per 1000 PAR) in 2007. The program goal was shifted toward elimination in 2009, the year when only 11 indigenous cases occurred. As the malaria burden declined, the dominant parasite species shifted from Plasmodium falciparum (Pf) to Plasmodium vivax (Pv), with *Pf* accounting for 76% of cases in 2006 but only 24% in 2009 (M. Rebueno-Trudeau, personal communication, July 8, 2012).

Yearly program expenditures were captured for the last two years of CLM (2007 and 2008) and the first year of reorientation for elimination (2009). Total expenditures, expenditures per PAR per year (PPY), and funding and staffing allocations are summarized in Table 3. Expenditures declined each year from a high of US$7.21 PPY in 2007 to US$3.28 PPY in 2009. While the GFATM accounted for the vast majority of spent funds, LGU yearly contributions also increased, as cost sharing from municipalities was required by GFATM. Provincial and national contributions were minimal and went toward personnel. Spending on diagnosis and treatment declined each year as RDTs and the retreatment of older ITNs were discontinued, and spending on personnel increased in 2009 when the program was reoriented toward elimination.

**Table 3:** Malaria Expenditures, Population at Risk, Cases, Funding Sources, Activities, and Personnel Time Across Study Provinces.

		Apayao				Laguna				Cavite		Benguet	
	2007	2008	2009	2006	2007	2008	2009	2010	1998	2000	2007	2004	2008
Program phase^1^	CLM	CLM	E	E	E/OB	E/OB	E	E	CLM	CLM/E	POR	E	POR
Population at risk^2^	103,633	106,642	109,742	8,532	8,760	8,995	9,236	8,892	9,814	13,101	11,033	5,841	6,325
Indigenous malaria cases^3^	246	35	11	3	256	9	4	7	24	3	0	0	N/A
Imported malaria cases^3^	0	0	0	2	4	N/A	N/A	N/A	22	1	3	12	N/A
Total expenditures^4^	$747,368	$570,603	$360,114	$27,844	$103,537	$110,093	$40,699	$43,562	$42,484	$23,575	$6,986	$16,185	$15,931
Expenditures per PAR^4^	$7.21	$5.35	$3.28	$3.26	$11.82	$12.24	$4.41	$4.90	$4.33	$1.81	$0.63	$2.77	$2.52
Funding sources													
Local government	14.9%	16.5%	23.9%	78.8%	42.2%	33.2%	69.7%	49.6%	29.7%	34.4%	59.1%	49.5%	48.2%
Provincial government	3.9%	4.1%	5.5%	7.8%	18.9%	11.3%	13.3%	31.5%	<1%	<1%	15.4	18.3%	15.2%
National government	1.7%	1.8%	2.5%	13.1%	38.8%	55.4%	16.8%	18.9%	70.3%	65.5%	25.6%	26.4%	26.7%
Global Fund	79.5%	77.4%	68.0%	-	-	-	-	-	-	-	-	-	-
Other	<1%	<1%	<1%	<1%	<1%	<1%	<1%	<1%	-	-	-	5.8%	9.8%
Malaria activities													
Diagnosis & Treatment	18.3%	12.6%	13.2%	12.5%	15.7%	7.1%	12.7%	10.6%	7.8%	4.1%	<1%	14.2%	16.5%
Prev & Vector Control	50.8%	41.6%	40.5%	26.2%	52.2%	66.6%	32.5%	45.8%	28.1%	30.4%	-	3.4%	3.5%
Surveillance	4.2%	4.6%	5.7%	15.3%	8.4%	5.7%	14.7%	8.7%	36.2%	43.7%	20.1%	46.6%	46.7%
IEC^5^	5.9%	7.6%	7.7%	8.8%	6.7%	5.5%	8.5%	5.5%	6.6%	13.1%	48.6%	21.4%	21.2%
Management/M&E^6^	20.8%	33.7%	32.9%	37.2%	17.0%	15.2%	31.8%	29.4%	21.4%	8.7%	31.3%	14.4%	12.1%
Personnel													
% of total expenditures	20.3%	24.3%	31.8%	95.2%	59.1%	46.3%	85.2%	63.4%	88.7%	79.8%	100.0%	91.4%	90.2%
Count	123	121	16	28	61	36	34	33	13	11	12	29	30
FTE equivalent^7^	33.33	30.66	8.25	5.68	14.40	11.29	6.70	4.33	5.86	3.47	1.04	2.85	2.86
Staffing ratio^8^	3.69	3.95	1.94	4.93	4.24	3.19	5.07	7.62	2.22	3.17	11.59	10.18	10.49

1 CLM = controlled low-endemic malaria; E = elimination; OB = outbreak; POR = prevention of reintroduction.

2 Population at risk (PAR) is defined as the total population residing in endemic *barangays*/municipalities. For Apayao, all municipalities are endemic. For Laguna, the endemic municipalities are San Gregorio, *Ala* *minos* ; Santiago II, San Pablo City; Bautista,and San Pablo City. For Benguet, the endemic municipalities are Itogon, Sablan, and Tuba; PAR figures for 2008 are projected from 2004 PAR based on the provincial population growth rate as none of the population are considered at risk after malaria-free certification. For Cavite, the endemic municipalities are Maragondon, Silang and Ternate.

3 N/A = No applicable records were found.

4 All expenditures are adjusted to 2010 USD.

5 IEC = Information and Education Campaign

6 M & E = Monitoring and Evaluation

7 Total time allocation for all malaria personnel are converted to the equivalent hours of a full-time employee (FTE).

8 The staffing ratio is derived by dividing the personnel count by the FTE equivalent.

**Figure 4:**
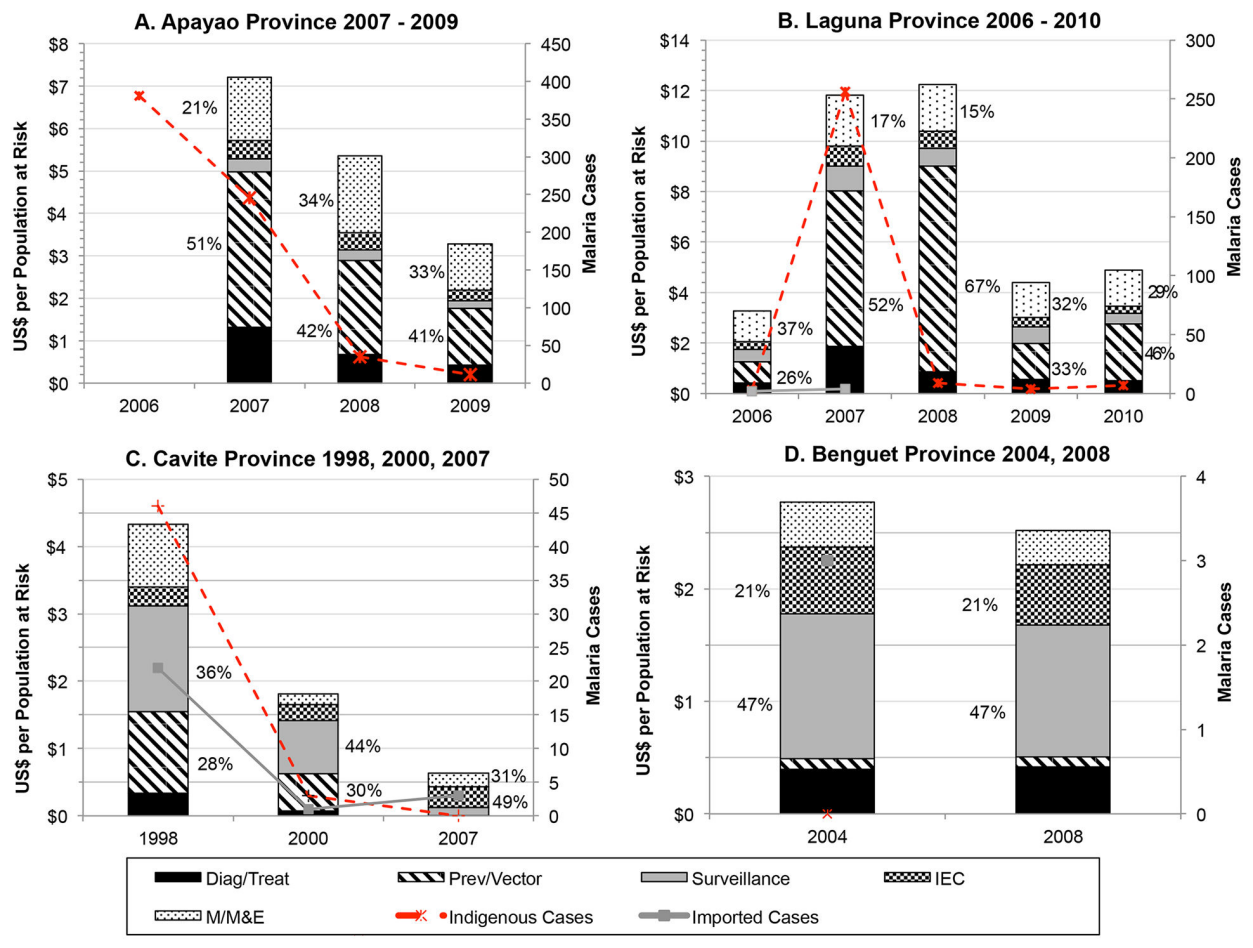
Malaria Program Expenditures for Selected Provinces of the Philippines. Total program expenditures by type of activity are displayed for each selected province for selected years, along with the number of indigenous and imported (when available) cases. The percent allocation for the two largest spending categories are given next to each bar. Notes: M/M&E = Management and Monitoring and Evaluation; IEC = Information and education campaign; Prev/Vector = Prevention and vector control; Diag/Treat = Diagnosis and treatment.

Overall, vector control dominated program spending. Figure 4, Panel A displays yearly spending levels and proportions across program activities. In 2007, 51% of all expenditures went toward prevention and vector control (i.e. ITN retreatment, LLIN distribution, and biannual IRS), declining to an average of 41% across 2008 and 2009 as LLIN coverage reached 100% in 2009, and ITN retreatment and IRS were scaled down. Correspondingly, spending on consumables declined (from 63% in 2007 to 50% in 2008/2009) and spending on M/M&E increased from 21% in 2007 to above 30% in later years.

### Laguna province

Before economic development and urbanization began in Laguna, malaria was endemic throughout the province. Indigenous cases numbered in the thousands and epidemic peaks occurred biennially in the late 1980s (see Figure 3, Panel B). During this time, vector control measures were sporadic, primarily used only in response to a rise in cases. Industrialization began in the 1990s, and waterways near construction areas became polluted. Key informants cited this as a potential cause for the reduction of mosquito-breeding habitats, followed by a sharp drop in annual cases. Continued development, along with focal vector control activities and prompt case management, helped to further reduce indigenous cases to zero in the early 2000s when malaria activities were discontinued and nearly all malaria program staff were reassigned. Indigenous cases remained zero until three *Pv* infections were detected in 2006, followed by an outbreak of 256 cases, the majority due to *Pf* (59%) in 2007 (G. Dela Cruz & R. Palos, personal communication, July 24, 2012).

Yearly program expenditures were captured from 2006 to 2010 (Figure 3, Panel B). Expenditures of US$3.26 PPY in 2006 mainly went toward personnel time on M/M&E (37%) and vector control (26%) at sites where cases occurred, mainly financed by the responsible local health offices (see Table 3). Even though implementation duties had been devolved, local program personnel lacked experience and training to manage the outbreak in 2007. Expenditures rose to US$11.82 PPY in 2007 and peaked at US$12.24 PPY in 2008 when the outbreak was contained, corresponding to increased resources from the national government to bring in regional malaria employees (extensions of the DOH) to lead and train local staff. The greatest share of spending went toward IRS and ITN distribution for outbreak containment (52% in 2007 and 67% in 2008). By 2008, 100% coverage among the population at risk for IEC and IRS in endemic areas was achieved and 100% coverage of LLINs distribution was reached in 2010. Trained local staff assumed all duties and the focus shifted to surveillance and maintaining zero indigenous cases to achieve malaria-free certification as evidenced by personnel spending as the largest share of program outlays (63%).

### Cavite province

Indigenous malaria caseload in Cavite has historically been low, confined to three endemic municipalities (Maragondon, Ternate, and Silang). According to available records, the highest number of indigenous cases, comprised of both *Pf* and *Pv* (77), was reported in 1987, declining to about 20 in the late 1990s (see Figure 3, Panel C), with *Pv* responsible for the last documented cases from 1998 to 2000 [10,12]. The proximity to Metro Manila has spurred urbanization since 1998 [11,12] and construction has polluted waterways and reduced vector breeding sites (primarily for *Anopheles* *flavirostris* ) [11]. In 2001, indigenous and imported cases plummeted to zero and remained at zero through to certification of malaria-free status in 2006. Since endemicity was naturally low, the few activities of the malaria program were run primarily by regional malaria personnel and never formally devolved.

Total expenditures declined for each selected year—from US$4.33 PPY in 1998 when the program was in the CLM phase, to US$1.81 PPY in 2000 when cases further declined to levels corresponding to elimination, to US$0.63 PPY in 2007, the first year of POR. Before malaria-free certification, regional staff conducted nearly all activities and funding mainly came from DOH (see Table 3; 70% in 1998, 66% in 2000). According to available program coverage indicators, IRS and ITN distribution and retreatment reached 100% coverage by 1998. Surveillance activities, the highest share of spending in 1998 (36%) and 2000 (44%), included passive and active case detection and mass blood surveys in suspected high transmission areas (e.g. construction sites and military camps that employed transient workers from other endemic provinces). LGUs provided salary support for midwives and health officers trained to collect blood smears. By 2007, nearly all spending went toward personnel with 59% coming from LGUs and only 26% from the national government. After malaria-free certification, all former regional malaria personnel were reassigned, retaining only minimal malaria M/M&E responsibilities for overseeing case detection (31% of total) while midwives in LGUs continued malaria IEC integrated with dengue (49%).

### Benguet province

Available records indicate that Benguet has had three endemic municipalities (Itogon, Tuba and Sablan) since the early 1990s (see Figure 3, Panel D). The last indigenous cases, all caused by *Pv*, occurred in 1992 (API=0.4 per 1000 PAR); however, resumption of local transmission is a possibility due to imported cases among migrant agricultural and mining workers from nearby endemic provinces (Local Government of Benguet 2012). NMCP records show that 98 imported cases were detected in 1992, falling to under 20 in the early 2000s; malaria records were not kept after Benguet was certified malaria-free in 2005 [13] (S. Puyao, personal communication, October 28, 2012).

Expenditure records were compiled for 2004, the year prior to certification, and 2008, representative of ongoing POR. In both years, no indigenous cases were detected and total program expenditures were constant: US$2.77 PPY in 2004 and US$2.52 PPY in 2008 (see Table 3). According to key informants, ongoing surveillance (47%) is considered vital for maintaining malaria-free status since vectors are still present according to entomological surveys [13]. Personnel at LGUs and the Provincial Health Team Office (PHTO) are integrated into other vector-borne or infectious disease programs. In both selected years, the 90% of spending on personnel supports 30 individuals working on malaria each year; each person spends an average of 10% of their time on malaria-specific activities (see Table 3). Because of rough terrain and poor infrastructure, the program heavily relies on local health offices for surveillance and disease-integrated IEC (21%).

### Looking across provinces and phases

Supplemented with key informant responses and literature review, a qualitative analysis was conducted to compare program expenditures across provinces. Several factors emerged to help explain the relative program spending levels and proportions across provinces and phases: differences in malaria burden and epidemiology, geography and level of economic development, sources of financing, organizational structure and capacity, and the degree to which malaria elimination was an active pursuit rather than a passive result of external factors and continued malaria control efforts. These characteristics are summarized in Table 4.

**Table 4:** Summary characteristics of selected provinces.

	Apayao	Benguet	Cavite	Laguna
Environment and ecology	Minimal infrastructure and mountainous terrain limits access to high-risk groups.	Mountainous terrain and remote municipalities are challenges for service delivery.	Rapid industrialization and development has reduced/polluted breeding sties.	Widespread urbanization and economic development eliminated breeding places.
	Prevalence of mining and logging in forested areas increase worker vulnerability.	Continued presence of vectors contributes to receptivity.	Primary and secondary vectors still thrive in areas with clear slow flowing streams.	Endemic areas are less-developed, remote, and ecology favors mosquito breeding.
Malaria epidemiology	Entire province is highly endemic.	Higher elevation, cooler climate contribute to lower transmission and receptivity.	Few endemic areas.	Entire province was endemic prior to industrialization in the early 1990s.
	Transmission cycle broken in mid-2000s through intense prevention and vector control.	Threat to POR of importation from neighboring endemic municipalities.	Threat to POR from migrant workers and military camps.	Outbreak cases were limited to specific rural areas.
				Zero indigenous cases since 2010.
Strategies and intervention choice	Scale-up of all activities with support of external funding.	Emphases on IEC and surveillance for elimination and POR	Emphasized IEC, surveillance for elimination and POR	100% coverage of IRS and LLINs in 2009 & 2010, active case detection in areas affected by outbreak.
	100% LLIN coverage achieved in 2008; reoriented toward elimination in 2009.		100% IRS, ITN distribution/retreatment in target areas by 1998.	
	Continuation of activities at reduced levels in more targeted areas as cases decline.	Personnel for ongoing surveillance, IEC and M/M&E activities retained.	Personnel for ongoing IEC, M/M&E and surveillance activities retained.	IRS and active surveillance discontinued when cases and resources declined post-outbreak.
Funding resources	GFATM grants provide the majority of funds while LGUs contribute an increasing share.	Domestically financed; rely on emergency allocation if outbreak occurred.	Domestically financed; rely on regional office if outbreak occurred.	LGUs provided majority of funds; national contributions increased during outbreak response.
	LGUs will need to support most activities when GFATM grants expire in 2014.	LGU’s provided majority of funds during elimination and POR for surveillance and integrated IEC by local health workers.	Significant funding by national during CLM and elimination, but shifted to LGUs for POR.	Domestically funded, requiring efficiency and narrow targeting of interventions.
Program structure and leadership	Devolved program led by PHO staff, implemented by MHOs with extensive NGO technical assistance.	Devolution had little effect on malaria activities since zero indigenous cases already achieved at that time.	Regional staff led malaria activities.	Devolved program managed by local health staff under supervision of provincial malaria coordinator.
	Minimal involvement of regional/national staff.	PHO staff continued to provide technical assistance and supported municipal health offices	Technical capacity retained through devolution.	Outbreak response required leadership and supervision by regional experts.

Note: CLM = controlled low-endemic malaria; GFATM = Global Fund to Fight AIDS, TB, and Malaria; IEC = information and education campaign; IRS = indoor residual spraying; LGU = local government unit; LLIN = long-lasting insecticide treated net; MHO = Municipal health office; PHO = Provincial health office; POR = prevention of reintroduction.

With the exception of outbreak in Laguna, program expenditures PPY decline through each phase of CLM, elimination, and POR. Among selected CLM years, expenditures PPY were higher in Apayao than in Cavite, commensurate with a higher malaria burden in Apayao, ecology hospitable to mosquito vectors, little economic development and infrastructure, and more diverse ethnic groups that require tailored interventions. For example, indigenous groups, such as the Aeta, are highly mobile and live in temporary structures with partial walls, making regular IRS and ITN/LLIN distribution difficult, and IEC must be customized for different dialects and low literacy. Higher spending levels in Apayao may also reflect larger resources provided by GFATM grants that enable purchasing of more capital equipment, but that also require more donor-specific reporting. Further, a heavier emphasis on commodity purchases accounts for the large share of spending for prevention and vector control (51% in 2007; 42% in 2008) compared to surveillance (6% in 2007; 8% in 2008). The program in Apayao also illustrates the importance of LGU political support: while donor funds enabled the program to formally devolve and rapidly scale-up, it also created resentment among LGU leaders that undermined implementation until the arrival of a new, supportive governor. In contrast, the program in Cavite was wholly domestically financed, and lower expenditures may also reflect spending for minimum necessities. For example, most equipment were beyond ‘useful life years’ as defined by accounting standards [14] and shared across multiple health programs. Commodities accounted for a lower proportion due to a greater emphasis on surveillance (36%) over prevention and vector control (28%). Moreover, because the program in Cavite was never formally devolved, no additional resources for training LGU staff were needed.

Expenditures PPY during the elimination phase are fairly comparable between those observed for Laguna in 2006 and for Apayao in 2009, years in which only a handful of indigenous cases were identified in both provinces. Although Laguna has experienced more industrialization than Apayao, border areas in Laguna where cases occurred in 2006 and in 2007-2008 outbreak are similarly difficult to access where marginalized groups reside (i.e. militia camps). In elimination years after the outbreak in Laguna, spending levels and allocations returned to pre-outbreak levels (US$3.26 PPY in 2006 compared to US$4.41 and US$4.90 PPY in 2009 and 2010, respectively). As both provinces are actively pursuing elimination, spending on prevention and vector control is larger than on surveillance activities.

The events in Laguna further provide an example of the relative resources required to stem and manage an outbreak (2007-2008). Expenditures PPY increased four-fold during outbreak years. In the absence of cases, the gap in expertise left when regional malaria staff were released in 2000 was not immediately felt. However, when neighboring provinces received GFATM support in 2006, all provinces in the region were formally devolved, including non-recipient provinces, leaving malaria program responsibilities in the hands of inexperienced LGU staff in Laguna. When the outbreak occurred, this human resource vacuum was filled by recruiting former regional workers and rapidly training local staff, largely financed by the national government. Due to resource constraints post-outbreak, IRS was discontinued in 2009 and active surveillance in 2010, with only “essential” staff sent for malaria trainings—a confluence of factors similar to those that precipitated the outbreak.

In sampled POR years, expenditures in Benguet were over four times as high as those in Cavite. While both provinces have similarly low receptivity for malaria transmission, the cost of delivering IEC and surveillance services in Benguet is likely greater due to accessibility challenges in reaching remote areas where migrants from malaria endemic provinces work. Key informants in both provinces (and in Laguna) stated, however, that greater resources are needed to improve outbreak response measures, increase surveillance vigilance along border areas, and maintain local malaria capacity. Without LGU financial reserves, emergency response may be hindered should cases be detected, and programs must rely on support from regional offices.

Thus, an adequate knowledge and capacity among program personnel are key components of successful malaria programs. Across phase transitions, the proportion of spending on personnel increased. Whereas only 20% of total program expenses went toward personnel in Apayao during the earliest control year (i.e. 2007), it increases to 32% by 2009. In post-outbreak elimination years in Laguna, this percentage is over 60%. During elimination and POR years, personnel expenditures ranged between 80 and 100% in Cavite and Benguet. Greater spending on personnel also reflects the need for robust surveillance activities that rely heavily on the skills of front-line health workers for case detection.

**Figure 5:**
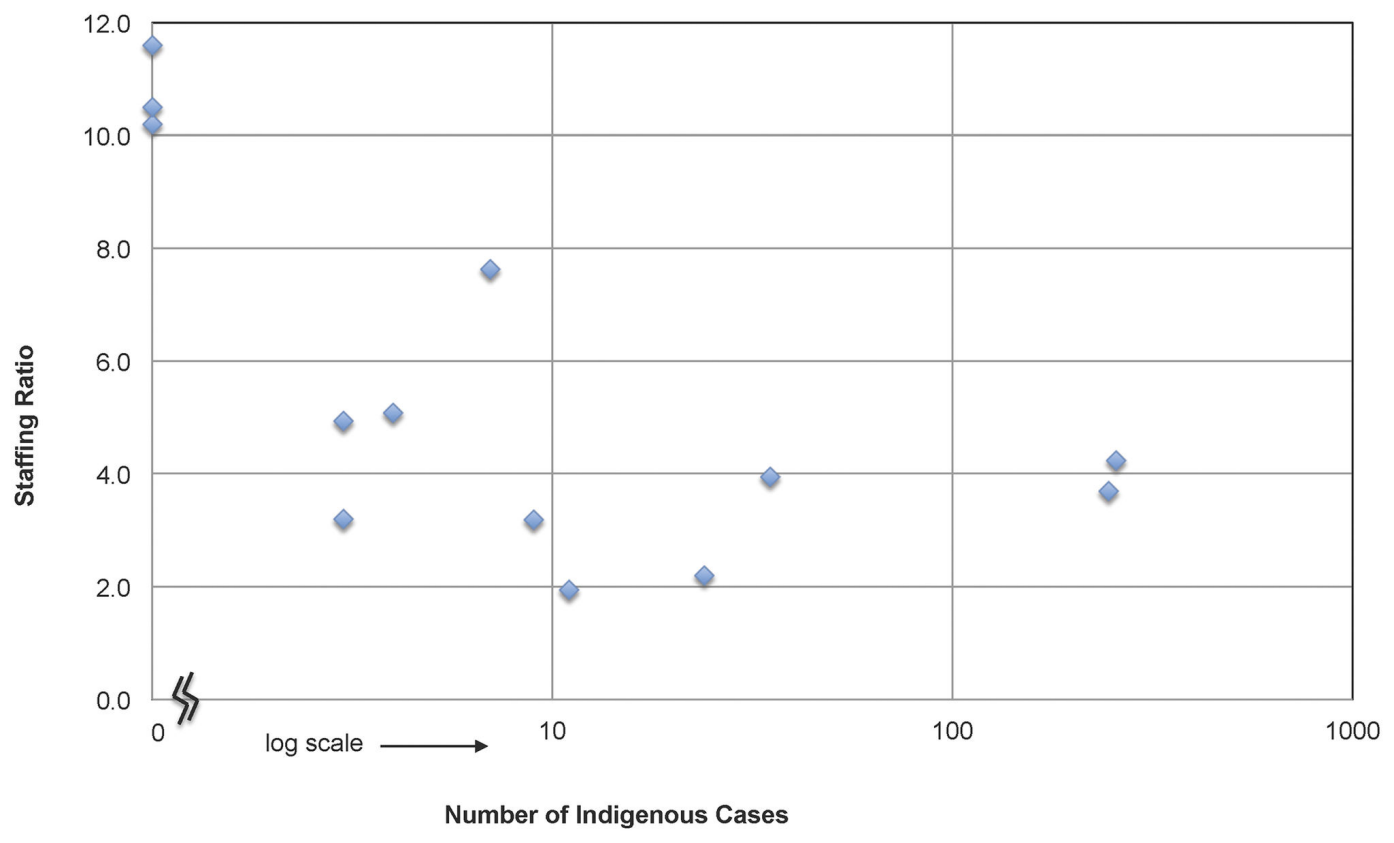
Staffing Ratios by Indigenous Caseload. Staffing ratios are calculated by dividing the raw count of personnel by the full-time equivalent (FTE) of the sum of all personnel time spent on malaria (i.e. staffing ratio = number of reported people working on malaria / number needed if all employees were working full time). A staffing ratio is calculated for each province-year observation and plotted according to the logged number of indigenous cases if the number of cases is greater than zero. For province-years where zero indigenous cases are recorded, points are plotted on the vertical axis itself.

Across phases, there are also distinct trends in staffing numbers and time allocation as cases decline (see Table 3). As expected, there is an overall decline in staff time and numbers allocated to malaria across phases. Notably, however, there is also a decline in staff ratios. Figure 5 plots the staffing ratios against the logged number of indigenous cases for each province-year data point. During control years, staffing ratios are low, reflecting relatively fewer numbers of staff that each spends a larger percentage of time on malaria. As the number of indigenous cases declines, the staffing ratio also declines and there is a move toward having relatively larger numbers of staff each spending small percentages of time on malaria. These staffing ratios may also reflect increased integration of malaria into other disease programs. For CLM program years, workers spent an average of 32% of their time on malaria, which declines to 26% for non-outbreak, elimination program years, and further to 9% for POR program years.

## Discussion

To assist policymakers and program managers in planning for malaria elimination, we sought to estimate the yearly resources that malaria programs in four selected provinces—Apayao, Benguet, Cavite, and Laguna—used during different phases of elimination. With the exception of outbreak in Laguna, overall expenditures PPY decrease in each phase from CLM to elimination to POR. Expenditures during CLM phases ranged from US$1.81 to US$7.21 PPY. Elimination phase expenditures ranged from US$2.77 to US$12.24 PPY, with the latter figure associated with the management of an outbreak. Provinces that are currently malaria-free and that did not have a deliberate goal to eliminate spent the least overall across all phases. In these eliminated provinces, expenditures associated with POR ranged from US$0.63 to US$2.77 PPY.

Qualitatively, variations in expenditures PPY across provinces appear to be associated with differences in geography and level of economic development, programs’ sources of financing, their organizational structure and capacity, and the degree to which malaria elimination is actively pursued (see Table 4). The costs of service delivery partially reflect the additional resources needed to reach remote and less developed areas. Expenditures PPY for POR are nearly four times higher in Benguet, when compared to Cavite, related to surveillance and IEC efforts that must reach migrant workers in distant areas bordering endemic neighbor provinces. Similarly, local housing styles and language barriers, such as those among Apayao’s ethnic groups, challenge IRS, ITN/LLIN, and IEC roll out, substantially increasing costs. Other studies find similar trends. For example, malaria expenditures for Vanuatu and the Solomon Islands, when adjusted by a remoteness and incapacity index (which accounts for population dispersion, travel distance, time, and accessibility) decline by a factor of 31 and 14, respectively [15]. Thus, program planning should account for greater resource needs for reaching vulnerable populations located in remote areas, rather than applying blanket formulas based on per-person unit costs.

Even though devolution of the malaria program responsibilities to LGUs was officially launched in 1993, the process and timeline for transferring duties varied across provinces. This process, while presenting opportunities for LGUs to take on greater leadership, exposed programs to greater risk if local staff were not adequately equipped to take on additional tasks. LGU capacity in Apayao was increased with additional resources from GFATM grants to support personnel training and formally devolve the malaria program. However, because GFATM assistance also triggered devolution in neighboring non-funded provinces, the risks associated with the lack of LGU capacity under a devolved organization was exposed in Laguna’s 2007 outbreak. These experiences highlight the need to build and maintain strong leadership and expertise within provincial malaria programs in a devolved system.

While donor assistance can add much-needed resources, it may also influence strategy and intervention choices, which may not necessarily be most efficient. The experience in Apayao shows that, even with large external financing, the transition between CLM and elimination can be made with decreasing marginal costs. However, this may not be the norm for other situations. For example, the Swaziland government spent an average of $4.57 PPY during CLM (2004-2008), but budgeted amounts for elimination rose dramatically to $12.88 PPY (2009-2013), a transition that is accompanied by an increase in external financing from 32% to 71% [16]. In both places, the influx of GFATM funding is accompanied by relatively greater spending on commodities for prevention and vector control and less on surveillance activities. In contrast, the program in Cavite, which was nearly all domestically financed during years when malaria was declining, spent relatively more on surveillance compared to vector control and still achieved and maintains zero indigenous cases.

Generally, as indigenous cases decline, the need for vector control and prevention activities decreases and there is greater emphasis on surveillance and IEC [1,17] as observed in all study provinces. In particular, in both Laguna and Apayao, spending on vector control dramatically reduces after 100% coverage of LLINs for targeted populations is reached and spending is largely comprised of personnel expenses for surveillance, IEC, and M/M&E. In addition, there is a general trend toward leveraging more numbers of personnel each working fewer hours on malaria activities during the later stages of elimination and POR. This staff allocation may reflect the need to have greater awareness and vigilance among larger numbers of public health personnel, who each spend a small amount of time monitoring the malaria situation. Wider coverage may also mean that cases can be detected more readily, such as through barangay health workers stationed in high risk areas.

Although there has been much attention paid to scaling up malaria control efforts, few studies have shown the financial resources required for malaria elimination. A handful of estimates are available for experiences during the Global Malaria Eradication Programme (1955-1970) (for a review, see 18), but it is unclear how relevant these are to the current environment as malaria burden has changed and new tools and challenges have surfaced [1,19]. Recently, a model simulation of program costs from five places (Hainan and Jiangsu provinces in China, Mauritius, Swaziland, and Zanzibar) shows that the cumulative costs for elimination is equal to or higher than those for maintaining malaria as very low levels indefinitely [16]. While this study was not able to document cumulative costs across phases, selected PPY expenditures show consistent declining yearly marginal costs across phases (with the exception of outbreak situations), corroborating findings from similar case studies. Program expenditures from Mauritius show that costs declined from US$5.39 to US$2.80 PPY during elimination, and further to US$2.09 PPY for POR [20]. Total expenditures in two districts in Sri Lanka for years representing later stages of malaria control (i.e moving from a high level of control to a state of CLM) also showed declining or stable expenditures: in 2004 and 2008, costs in Anuradhapura declined from US$1.57 to US$0.81 PPY, respectively, but costs in Kurunegala were constant at US$1.94 and US$1.84 PPY, respectively [21].

### Limitations

With the retrospective data capture, some cost components are estimated based on key informant input and standard commodity unit costs where expenditure records were not available. Thus, reported expenditure levels are only indicative of the magnitude of true costs. Additionally, full economic costs are not captured, but private direct and indirect costs are likely to be very small due to the small number of cases occurring during elimination. Lastly, we do not look at early years when initial malaria interventions are first implemented or scaled up in efforts to bring rampant malaria under control, which can entail large amounts of financing and are necessary before elimination can even occur.

It is unclear how generalizable these findings are to other provinces in the Philippines as a purposeful sampling frame was used and data availability was a deciding factor in selecting sampled years. As such, expenditure data are meant to describe general trends that can be understood from specific illustrative examples rather than being used for budgeting purposes. Similarly, results may not be generalizable to other countries. Expenditures are standardized by PAR, but each country may calculate PAR differently. Moreover, the pace of economic development in relation to malaria program implementation may moderate the speed at which marginal costs may decline over time. This appeared to be advantageous for some of the study provinces and limits the degree to which conclusions can be applied to places developing at a slower pace. Lastly, countries such as the Philippines that have declared a national goal of malaria elimination may have other advantages that lower costs compared to countries where elimination may not be feasible and prohibitively costly (e.g. Zanzibar, see 22).

However, the strengths, weaknesses, and experiences of the selected provinces in the Philippines may provide country program managers with insight in how to deal with similar challenges and adequately plan for such situations in their local context. Understanding how programs spend and allocate resources, and the factors that moderate these choices, may inform strategic planning and resource mobilization, particularly in deciding which activities should be supported and how to provide the necessary tools and personnel to ensure program integrity.

While cost sharing and efficiency spending are desirable, this case study of selected provinces in the Philippines indicates there is a real risk that outbreaks may occur and may not be effectively managed if there are large resource gaps—particularly in available staff experience and expertise—that may exist in a devolved health care system. As the GFATM grant to the Philippines is set to finish in 2014, a concerted effort to build local capacity in preparation for this transition should be made to minimize risks due to program disruption or gaps in human resources. Based on the program histories of Laguna, Cavite, and Benguet in funding malaria activities in elimination settings, Apayao LGUs should be able to take on the financial responsibilities provided political will is sustained. If this is the case across many of the higher-burden GFATM-supported provinces, and if emerging cases of *P* *. knowlesi* cases can be controlled [23], then the Philippines would be well-positioned to continue to progressively eliminate malaria. Moreover, there are national plans to establish regional hubs that maintain stockpiles of equipment, drugs, and supplies and could coordinate emergency response for provinces that may not have the ability to quickly mobilize local resources [7]. Above all, maintaining a cadre of front-line workers who are trained on malaria appears to be necessary for keeping indigenous cases at or near zero. 

## Supporting Information

Appendix S1 **Additional details about the methods employed in this study are contained in Appendix S1.**
(DOCX)

## References

[1] Cotter C, Sturrock HJW, Hsiang MS, Liu J, Phillips AA et al. (2013) The changing epidemiology of malaria elimination: new strategies for new challenges. Lancet (2013). PubMed: 23594387 10.1016/S0140-6736(13)60310-4 PMC10583787 23594387

[2] Murray CJL, Rosenfeld LC, Lim SS, Andrews KG, Foreman KJ et al. (2012) Global malaria mortality between 1980 and 2010: a systematic analysis. Lancet 379(9814): 413-431. doi:10.1016/S0140-6736(12)60034-8. PubMed: 22305225. 22305225 10.1016/S0140-6736(12)60034-8

[3] World Health Organization (2007) Malaria elimination - A field manual for low and moderate endemic countries. Geneva: World Health Organization.

[4] Back Roll Malaria Partnership . (2008) The Global Malaria Action Plan: for a malaria-free world (2008). Geneva: World Health Organization.

[5] Cohen JM, Moonen B, Snow RW, Smith DL (2010) How absolute is zero? An evaluation of historical and current definitions of malaria elimination. Malar J 9: 213. doi:10.1186/1475-2875-9-213. PubMed: 20649972. 20649972 10.1186/1475-2875-9-213 PMC2983111

[6] Republic of the Philippines Department of Health (2012) Malaria Cases and Deaths. Manila: National Malaria Control Program.

[7] Republic of the Philippines Department of Health (2011). pp. 2011-2016. Malaria Medium Term Development Plan. Manila: National Malaria Control Program.

[8] Espino F, Beltran M, Carisma B (2004) Malaria control through municipalities in the Philippines: struggling with the mandate of decentralized health programme management. Int J Health Plan Mgmt 19: S155-S166. doi:10.1002/hpm.782. PubMed: 15686067. 10.1002/hpm.782 15686067

[9] The World Bank. (2012) World Development Indicators 2012: Philippines. Washington, DC: The World Bank http://data.worldbank.org/country/philippines. Accessed: 4 November 2012.

[10] Republic of the Philippines Department of Health (2005) Evaluation of the malaria situation in the province of Cavite. Manila: National Malaria Control Program.

[11] Isidoro M, Makoto Y (2007) Urbanization process and the changing agricultural landscape pattern in the urban fringe of Metro Manila, Philippines. Environment and Urbanization 19(1): 191-206. doi:10.1177/0956247807076782.

[12] Cavite Provincial Health Team Office (1998). Malaria Cases. Province of Cavite.

[13] Republic of the Philippines Department of Health (2004) Evaluation of the malaria situation in the province of Benguet. Manila: National Malaria Control Program.

[14] Internal Revenue Service (1987) REV-PROC, Accelerated cost recovery: Recovery classes: Class lives: Recovery periods.--, Revenue Procedure 87-56, (Jan. 01 1987). Washington DC: United States Government.

[15] Beaver C, Pontifex S, Zhao Y, Marston L, Bobogare A (2011) Application of a remoteness index: funding malaria programs. International Journal of Geoinformatics 7(1): 15.

[16] Sabot O, Cohen JM, Hsiang MS, Kahn JG, Basu S et al. (2010) Costs and financial feasibility of malaria elimination. Lancet 376(9752): 1604-1615. doi:10.1016/S0140-6736(10)61355-4. PubMed: 21035839. 21035839 10.1016/S0140-6736(10)61355-4 PMC3044845

[17] Moonen B, Cohen JM, Snow RW, Slutsker L, Drakeley C et al. (2010) Operational strategies to achieve and maintain malaria elimination. The Lancet, Series: Malaria Elimination, 376: 1592-1603. PubMed: 21035841. 10.1016/S0140-6736(10)61269-X PMC3037542 21035841

[18] Mills A, Lubell Y, Hanson K (2008) Malaria eradication: the economic, financial and institutional challenge. Malar J 7: S11. doi:10.1186/1475-2875-7-11. PubMed: 19091035. 19091035 10.1186/1475-2875-7-S1-S11 PMC2604875

[19] Feachem RGA, Phillips AA, Hwang J, Cotter C, Wielgosz B et al. (2010) Shrinking the Malaria Map: progress and prospects. Lancet Series Malar Elimination 376: 1566-1578 PubMed: 21035842. 10.1016/S0140-6736(10)61270-6 PMC3044848 21035842

[20] Tatarsky A, Aboobakar S, Cohen JM, Gopee N, Bheecarry A et al. (2011) Preventing the reintroduction of malaria in Mauritius: a programmatic and financial assessment. PLOS ONE, 6: e23832. doi:10.1371/journal.pone.0023832. PubMed: 21912645. 10.1371/journal.pone.0023832 PMC3166284 21912645

[21] Abeyasinghe RR, Galappaththy GNL, Smith Gueye C, Kahn JG, Feachem RGA (2012) Malaria control and elimination in Sri Lanka: documenting progress and success factors in a conflict setting. PLOS ONE 7(8): e43162. doi:10.1371/journal.pone.0043162. PubMed: 22952642. 10.1371/journal.pone.0043162 PMC3430652 22952642

[22] Smith DL, Cohen JM, Moonen B, Tatem AJ, Sabot OJ et al. (2011) Solving the Sisyphean problem of malaria in Zanzibar. Science 332(6036): 1384-1385. doi:10.1126/science.1201398. PubMed: 21680828. 21680828 10.1126/science.1201398

[23] Luchavez J, Espino FE, Curameng P, Espina R, Bell D et al. (2008) Human infections with Plasmodium knowlesi, the Philippines. Emerg Infect Dis 14(5): 811–813. doi:10.3201/eid1405.071407. PubMed: 18439369. 18439369 10.3201/eid1405.071407 PMC2600254

