# Supplementary material for: Determinants of Malaria Program Expenditures during Elimination: Case Study Evidence from Select Provinces in the Philippines
Source: PLoS One. 2013 Sep 27;8(9):e73352. doi: 10.1371/journal.pone.0073352 (PMC3785467; doi:10.1371/journal.pone.0073352)
Supplement: Appendix S1 — Additional details about the methods employed in this study are contained in Appendix S1. (DOCX) [file pone.0073352.s001.docx]

**Appendix S1**

*Research team and reflexivity*

The following female authors (along with their credentials and positions at the time the research was underway) conducted the key informant interviews:

- AB (BA) is a graduate student in public health with prior experience in malaria programming;
- GN (MSc) is a research assistant with prior experience in interviewing techniques;
- LRE (MD) is an independent consultant with prior experience in GFATM program coordination in the Philippines; and
- CJC (RN) is the technical writer and coordinator for the Philippines DOH Infectious Disease Office.

All interviewers underwent a four-day training in Manila prior to the commencement of research activities, which included pre-testing of the interview guides. A two-week pilot was conducted in Sorsogon province where all interview guides, data tracking forms, and data processing procedures were practiced.

With assistance from the NMCP Director, interviewers were introduced to potential key informants at DOH regions offices, or Regional Centers for Health Development (CHD), for the selected provinces. The DOH also provided letters of introduction authorizing the research to take place and to facilitate introductions to local program offices. Researchers were instructed to introduce themselves and explain the objectives of the study to each potential study participant. For key informants who provided written informed consent to participate in the study, interviewers noted their current and former position in relation to the malaria program, any observations about interviewee non-verbal cues, and general interest in participating in the study. These factors were taken into account when reviewing interview transcripts.

*Study design*

The case study’s design was based on a grounded theory approach to elicit success factors and challenges that explain the evolution of the Philippines malaria control program. In this effort, financial resources was identified as one key dimension of understanding the constraints (or lack thereof) under which program choices were made. Key informants were purposefully selected based on current or past experience in working with local malaria programs in the selected provinces. Key informants also referred interviewers to other potential study participants at the conclusion of each interview. Potential study participants were either approached in-person if they were present in the health office or contacted through phone to set up a meeting time. Interviews were conducted in private settings in which only the interviewers and consenting participants were present.

Interviews were conducted at multiple levels in the public health system. At the national-level, participants included current and former coordinators, directors, and managers at the DOH, as well as managers at major NGO partners that work closely with the DOH to coordinate malaria activities. Interviews were further conducted at regional-level CHD offices, provincial health offices, and hospitals, municipal and *barangay* health offices as needed. These individuals included managers, field workers, drivers, temporary workers, nurses, and other program personnel to elicit information from multiple perspectives. Interviews were conducted in each province until researchers mutually agreed that data saturation had been reached. In total, 54 key informants were interviewed. At each office or site visit, additional data regarding program expenditures, epidemiological indicators, or intervention coverage were collected for the selected sample years.

Interviewers were instructed to follow two interview guides—one aimed at eliciting an in-depth understanding of the malaria program and key events, and one aimed at identifying specific expenditure data. Interview guides were developed by the Principal Investigators and pilot tested in Sorsogon province before data collection proceeded in the selected study provinces. Each interview was conducted by at least two researchers, at least one of which is fluent in Tagalog. Interviews were tape-recorded and de-identified audio files were sent for translation (if the interview was conducted in Tagalog) and transcription. Interviews lasted from 30 minutes to three hours, depending on the knowledge and experience of the participant. Written notes taken during the interview were then combined with transcriptions for later data analysis. Although transcripts were not returned to participants for any corrections, interviewers made corrections according to their notes and recollections.

*Analysis and findings*

A coding scheme was developed to categorize interview content into themes. Themes were initially identified in advance based on past research experience and the literature review. Additional themes were found after testing the proposed coding scheme. A final set of codes was used after an iterative process involving JL, CSG, GN, and AB through multiple rounds of practice coding, discussion, and agreement. All coding was performed using Atlas ti 6.2.

A preliminary presentation of the findings and expenditure data was given to a selected number of key informants at the conclusion of data collection at the DOH offices in Manila. Participants provided feedback and comment on the findings and directed researchers to additional sources where data gaps may be filled.

*Expenditure calculations*

Personnel expenditures reflect total compensation for each employee, including salary and associated benefits. Only standard benefits in all provinces and at all levels of government were captured; more specific benefits like insurance withholdings, employer matches for social security, etc were excluded as such information was not available in all locations. The value of in-kind labor provided by community volunteers, including *barangay* health workers, was estimated based on average honorarium payments.

Expenditures for consumables used in diagnosis and treatment were calculated based on a standard formula of supplies required to perform one blood smear at prevailing purchase prices and the number of blood smears conducted. Drug quantities were calculated based on standard treatment protocols, the number of positive cases, and the infection type on record. When exact patient ages were not available, average drug quantities for the closest age group were used. For trainings conducted by the national government, standard per diem benefits were assumed when actual travel reimbursement records were missing. Travel for regular staff within provinces and the values of meals while in the field could not be estimated in the absence of records, possibly leading to estimates that are too conservative for services.

Expenditures for health office utilities and maintenance were universally unavailable, as malaria activities had already been integrated into overall vector-borne disease programs and costs were difficult to disaggregate. Expenditures for recurring maintenance and utilities are likely to be minimal for integrated programs, but nevertheless may result in an under-estimation of services. The estimated commercial value of real estate was not captured, as reliable estimates could not be obtained from key informants and records were not available at health offices. Values of capital equipment, including furniture, computers, microscopes, and vehicles, were estimated based on useful life years remaining and current resale value.

A sensitivity analysis was conducted to determine to what extent the assumptions made regarding missing data influence the total program expenditures. For personnel costs, employee benefits for social security employer contributions are missing for some program employees. An upper value of missing benefits was estimated at 10% of base salary, which is above the reported average employer contributions for social security in the Philippines (7.07% of monthly insured earnings^[[1]](#endnote-1)^). Increasing benefits by 10% had no impact on expenditures PPY and increased the share of expenditures for the Personnel category by less than 1%. The cost estimates for in-kind labor, drug quantities for average age groups, training per diems, and travel and meals for field staff all accounted for less than 1% of annual expenditures and thus, varying these estimates by as much as 100% did not have any measurable effect on total expenditure amounts. Most capital equipment used by programs was beyond useful life years and had negligible value. The cost of utilities, maintenance, and real estate were not available in any study provinces, thus they were universally excluded from our analysis and no assumptions were made as to their values.

1. Source: Social Security Programs Throughout the World: Asia and the Pacific, 2010 – Philippines. <http://www.socialsecurity.gov/policy/docs/progdesc/ssptw/2010-2011/asia/philippines.html>. Accessed June 27, 2013. [↑](#endnote-ref-1)
